# Baseline-Dependent Biological Response to Peri-Implant Soft Tissue Augmentation: A Secondary Analysis of a Randomized Clinical Trial

**DOI:** 10.3390/jfb17090439

**Published:** 2026-09-01

**Authors:** Jakub Hadzik, Paweł Kubasiewicz-Ross, Umberto Romeo, Krzysztof Kujawa, Nicola Alberto Valente, Marzena Dominiak

**Affiliations:** 1 Department of Dental Surgery, Faculty of Dentistry, Wroclaw Medical University, 50-425 Wroclaw, Poland; 2 Department of Oral and Maxillofacial Sciences, Faculty of Medicine and Dentistry, Sapienza University of Rome, 00161 Rome, Italy; 3 Statistical Analysis Centre, Wroclaw Medical University, 50-425 Wroclaw, Poland; 4 Division of Periodontology, School of Dental Medicine, Department of Surgical Sciences, Faculty of Medicine and Surgery, University of Cagliari, 09124 Cagliari, Italy

**Keywords:** peri-implant soft tissue, connective tissue graft, collagen matrix, soft tissue phenotype, ultrasonography, randomized clinical trial

## Abstract

Objectives: Clinical outcomes of peri-implant soft tissue augmentation remain variable, and baseline-associated factors of treatment response are poorly understood. This exploratory study aimed to evaluate baseline-associated patterns of peri-implant soft tissue gain following augmentation with connective tissue grafts (CTG) and volume-stable collagen matrices (VCMX). Materials and Methods: This study represents a secondary analysis of a randomized clinical trial including 32 implant sites (CTG, *n* = 16; VCMX, *n* = 16). Peri-implant soft tissue thickness (STT) was measured using high-frequency ultrasonography at baseline (T0), 3 months (T1), and 12 months (T2). Absolute and relative STT gains were analyzed, and their relationship with baseline thickness was assessed. Results: Both augmentation approaches resulted in significant increases in soft tissue thickness, with most of the gain occurring during the early healing phase. At 12 months, CTG demonstrated greater absolute tissue gain than VCMX. At the mid-buccal measurement site, mean relative gain was approximately 225% with CTG and 147% with VCMX. The strongest inverse correlation between baseline STT and relative gain was also observed at this site in the CTG group (*r* = −0.815, *p* < 0.001). Sites with thinner tissues showed greater proportional increases, whereas thicker tissues demonstrated a reduced relative response. Conclusions: Relative soft tissue gain was inversely associated with baseline soft tissue thickness. Thinner tissues exhibit greater proportional increases, whereas thicker tissues demonstrate a lower relative gain. These findings support a phenotype-informed and individualized approach to peri-implant soft tissue management.

## 1. Introduction

### 1.1. Clinical Relevance of Peri-Implant Soft Tissue Thickness

Peri-implant soft tissue thickness is a key determinant of peri-implant tissue stability and esthetic outcomes, influencing wound healing, marginal bone remodeling, and emergence profile development [1,2].

Thin peri-implant mucosa (≤2 mm) has been consistently associated with increased crestal bone remodeling, likely related to biological width establishment [1]. This threshold vertical peri-implant mucosal thickness is widely recognized in the literature and supported by consensus reports, indicating that reduced tissue thickness may represent a risk factor for peri-implant tissue instability [3,4,5,6].

The peri-implant soft tissue phenotype, including mucosal thickness and keratinized mucosa width, is therefore considered a site-specific risk indicator for peri-implant complications [3,7,8]. However, despite its recognized importance, the role of baseline soft tissue thickness in shaping the response to augmentation procedures is still not fully understood.

### 1.2. Soft Tissue Augmentation in Implant Dentistry

Autogenous connective tissue grafts (CTG) are widely regarded as the gold standard for peri-implant soft tissue augmentation due to their predictable clinical performance and long-term peri-implant tissue stability [2,3,9]. CTG effectively increases mucosal thickness and remains the reference approach in clinical practice.

However, autogenous grafting is associated with increased surgical complexity and donor site morbidity. To reduce treatment burden, xenogeneic collagen-based substitutes, including volume-stable collagen matrices, have been introduced as less invasive alternatives, providing clinically relevant soft tissue gain, although generally inferior to autogenous grafts [10,11].

Despite the availability of different augmentation modalities, clinical outcomes remain variable [12,13,14]. The magnitude of soft tissue gain is inconsistent, and baseline-associated predictors of treatment response are poorly defined. This highlights the need for a more individualized, phenotype-driven approach to soft tissue augmentation.

### 1.3. Unresolved Clinical Questions

Despite the widespread use of peri-implant soft tissue augmentation, treatment outcomes remain inconsistent [10,15]. The extent to which baseline soft tissue thickness determines the magnitude of tissue gain remains poorly understood, and the role of site-specific factors in soft tissue remodeling is unclear.

Identifying baseline-associated factors of tissue gain may be critical for improving treatment predictability and enabling more individualized therapeutic strategies.

Absolute gain expresses the change in soft tissue thickness in millimeters without accounting for the baseline value, whereas relative gain expresses the change in proportion to baseline thickness and may therefore reveal baseline-associated patterns not evident from absolute measurements alone.

We hypothesized that relative soft tissue gain may reflect a baseline-associated response pattern that is not captured by conventional absolute measurements.

### 1.4. Aim of the Study

The aim of this study was to evaluate baseline-associated patterns of peri-implant soft tissue response following augmentation, with particular emphasis on the relationship between baseline tissue thickness and both absolute and relative tissue gain.

## 2. Materials and Methods

### 2.1. Study Design

This study represents a secondary analysis of data derived from a prospectively conducted randomized clinical trial performed at a single academic center (Wroclaw Medical University, Poland). The primary outcomes of the original trial were described earlier by Hadzik and Dominiak [16].

The original trial compared peri-implant soft tissue augmentation using an autogenous connective tissue graft (CTG) and a volume-stable collagen matrix (VCMX), with implant sites randomly allocated to treatment groups.

The study protocol of the parent randomized clinical trial was approved by the Bioethics Committee at Wroclaw Medical University (approval no. KB-863/2021) and conducted in accordance with the Declaration of Helsinki. The parent RCT was registered at ClinicalTrials.gov registration number NCT07324187 [16].

The present analysis represents a secondary evaluation of the original dataset, addressing a different research question focused on baseline-associated factors related to treatment response and site-specific patterns of peri-implant soft tissue gain, and did not involve any additional medical interventions.

### 2.2. Study Population

The study population consisted of patients enrolled in a previously conducted randomized clinical trial evaluating peri-implant soft tissue augmentation in the aesthetic zone.

The present secondary analysis included 32 augmented implant sites (CTG, *n* = 16; VCMX, *n* = 16) contributed by 30 patients.

Randomization in the parent trial was performed at the implant-site level, and the augmentation material was assigned separately to each implant site. Therefore, when two implants were placed in the same patient, treatment allocation was performed separately for the two sites. These implant sites were located in different quadrants and separated by at least two teeth or were located in opposing jaws. Each implant therefore represented a spatially distinct surgical and measurement site and constituted the unit of randomization and analysis. As only two patients contributed more than one implant site, the potential effect of within-patient dependence cannot be excluded and was considered when interpreting the findings. Importantly, no patient contributed more than one implant site to the same treatment group; in both patients with two implant sites, the sites were allocated to different augmentation modalities.

Detailed inclusion and exclusion criteria have been reported previously. In brief, systemically healthy adult patients requiring single-tooth implant rehabilitation in the aesthetic zone (incisors, canines, or premolars) were included. Eligible sites presented baseline peri-implant soft tissue thickness ranging from 0.5 to 2.5 mm.

### 2.3. Augmentation Procedures

Peri-implant soft tissue augmentation procedures were performed according to a standardized protocol within the parent randomized clinical trial.

In that trial, sites were allocated to receive either an autogenous de-epithelialized connective tissue graft (CTG) or a volume-stable collagen matrix (VCMX; Fibro-Gide^®^, Geistlich Pharma AG, Wolhusen, Switzerland). The CTG or VCMX was positioned on the buccal aspect of the implant site to increase peri-implant soft tissue thickness.

For the present secondary analysis, only augmented sites were included. Accordingly, sites were categorized into two groups: G1 (CTG, *n* = 16) and G2 (VCMX, *n* = 16).

### 2.4. Soft Tissue Thickness Assessment

Peri-implant soft tissue thickness (STT) was assessed using high-frequency ultrasonography, a non-invasive and validated method for soft tissue measurements in implant dentistry [17,18,19].

**Figure 1:**
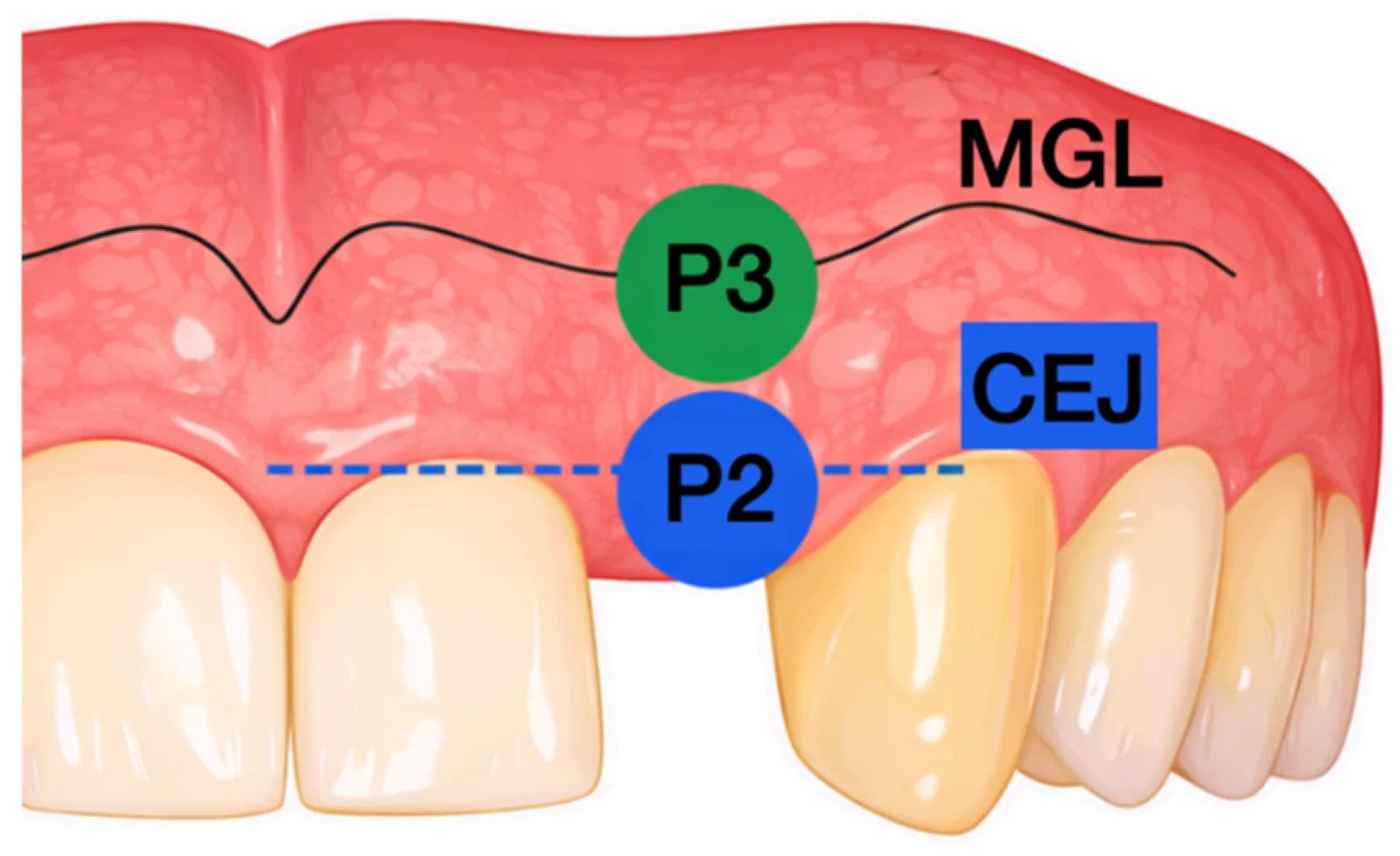
Schematic illustration of peri-implant soft tissue thickness (STT) measurement points.

Measurements were performed at two standardized buccal reference points along the implant axis: P2, located at the midline connecting the cemento-enamel junctions (CEJ) of adjacent teeth, and P3, located at the level of the mucogingival line (MGL), as previously described. Measurements were performed using the PIROP-G ultrasound biometric scanner (ECHOSON S.A., Puławy, Poland), equipped with a dedicated intraoral probe Figure 1.

Assessments were conducted at baseline (T0), 3-month early follow-up (T1), and 12-month late follow-up (T2). All measurements were performed by a single calibrated examiner. The measurement method, including examiner calibration and reliability assessment, was evaluated in a dedicated methodological study, which demonstrated high agreement with the clinical reference method [17].

### 2.5. Outcome Variables

The primary objective of the present analysis was to evaluate baseline-associated patterns of peri-implant soft tissue response. The primary outcome variable was relative soft tissue gain (ΔSTT_relative).

Absolute soft tissue gain (ΔSTT_absolute) was defined as the difference between the soft tissue thickness measured at follow-up and the corresponding baseline measurement. Relative soft tissue gain (ΔSTT_relative) was calculated by expressing this difference as a percentage of the baseline soft tissue thickness. Thus, the reported relative-gain values represent the percentage increase above baseline rather than the final thickness expressed as a percentage of baseline.

Baseline STT (T0) was analyzed as a continuous predictor variable. In addition, an exploratory categorical analysis was performed using a 1 mm threshold (≤1 mm vs. >1 mm) based on the periodontal phenotype classification proposed by Jepsen et al. [20]. This categorization was used solely to facilitate phenotype-based comparisons, whereas the primary analyses were performed using baseline STT as a continuous variable.

Outcome variables were evaluated separately for each measurement site (P2 and P3) and follow-up time point (T1 and T2). As an exploratory parameter, soft tissue augmentation efficiency was calculated for the 12-month interval by dividing the absolute tissue gain from T0 to T2 by graft thickness (efficiency = ΔSTT_T0–T2/graft thickness).

### 2.6. Statistical Analysis

Statistical analysis was performed using R software release R 4.5.2 (2025) (R Foundation for Statistical Computing, Vienna, Austria).

No formal a priori sample size calculation was performed, as the present study represents a secondary analysis of an existing randomized clinical trial dataset. The analysis was considered exploratory.

Continuous variables were summarized using means and standard deviations or medians, interquartile ranges, and ranges, as appropriate. Distributional assumptions were assessed using the Shapiro–Wilk test and graphical inspection of the data. Model assumptions were additionally evaluated using residual diagnostic plots and, where applicable, the Breusch–Pagan test.

Longitudinal STT measurements obtained at P2 and P3 at T0, T1, and T2 were analyzed using a linear mixed-effects model fitted by restricted maximum likelihood. Time, treatment group, measurement site, and their interactions were included as fixed effects, whereas implant ID was included as a random intercept to account for repeated measurements. Fixed-effect inference was based on Satterthwaite’s approximation. Within-group post hoc comparisons between time points used the Kenward–Roger degrees-of-freedom method and were adjusted using the Bonferroni procedure.

Associations between baseline STT and absolute and relative tissue gain from T0 to T2 were evaluated separately for each treatment group and measurement site using Pearson correlation coefficients. LOESS smoothing was used for exploratory visualization of potential nonlinear patterns.

The exploratory effects of treatment group, dichotomized baseline phenotype, and their interaction on absolute and relative gain were evaluated using the Scheirer–Ray–Hare test. Where computationally feasible, the findings were additionally examined using a robust two-way median-based procedure.

The difference in absolute T0–T2 gain between P2 and P3 was assessed using a paired-samples *t*-test after confirming approximate normality of the within-implant differences. Within the CTG group, associations between graft thickness and absolute or relative gain at P2 were evaluated using Pearson correlation coefficients. Augmentation-efficiency distributions were compared between CTG and VCMX separately at P2 and P3 using the Mann–Whitney U test.

Multivariable linear regression models were used to evaluate the adjusted associations of augmentation material, measurement site, and baseline STT with absolute and relative T0–T2 tissue gain. P2 and P3 were retained as separate site-specific observations because they represent anatomically distinct measurement locations, with measurement site included as an explanatory variable. Separate models were fitted for absolute and relative gain, with augmentation material, measurement site, and baseline STT included as explanatory variables. Model assumptions were evaluated using residual diagnostic plots and the Breusch–Pagan test. Because the residual distributions of the pooled models deviated from normality, median regression at the 50th percentile was additionally performed as a robust sensitivity analysis. These pooled multivariable analyses were considered exploratory.

Statistical significance was set at *p* < 0.05. Given the post hoc and exploratory nature of the study, no global adjustment for multiple comparisons was applied. Adjustment was used only for the explicitly indicated post hoc contrasts; the remaining *p*-values should therefore be interpreted as nominal.

## 3. Results

### 3.1. Baseline Characteristics

A total of 32 implant sites were included in the analysis, with sites allocated to two groups according to treatment modality (G1—CTG, *n* = 16; G2—VCMX, *n* = 16). Baseline characteristics relevant to the present secondary analysis are summarized in Table 1.

### 3.2. Temporal Dynamics of Soft Tissue Response

Both augmentation approaches resulted in measurable increases in peri-implant soft tissue thickness, as previously reported in the parent randomized clinical trial; therefore, the present analysis focuses on determinants of the observed biological response.

**Figure 2:**
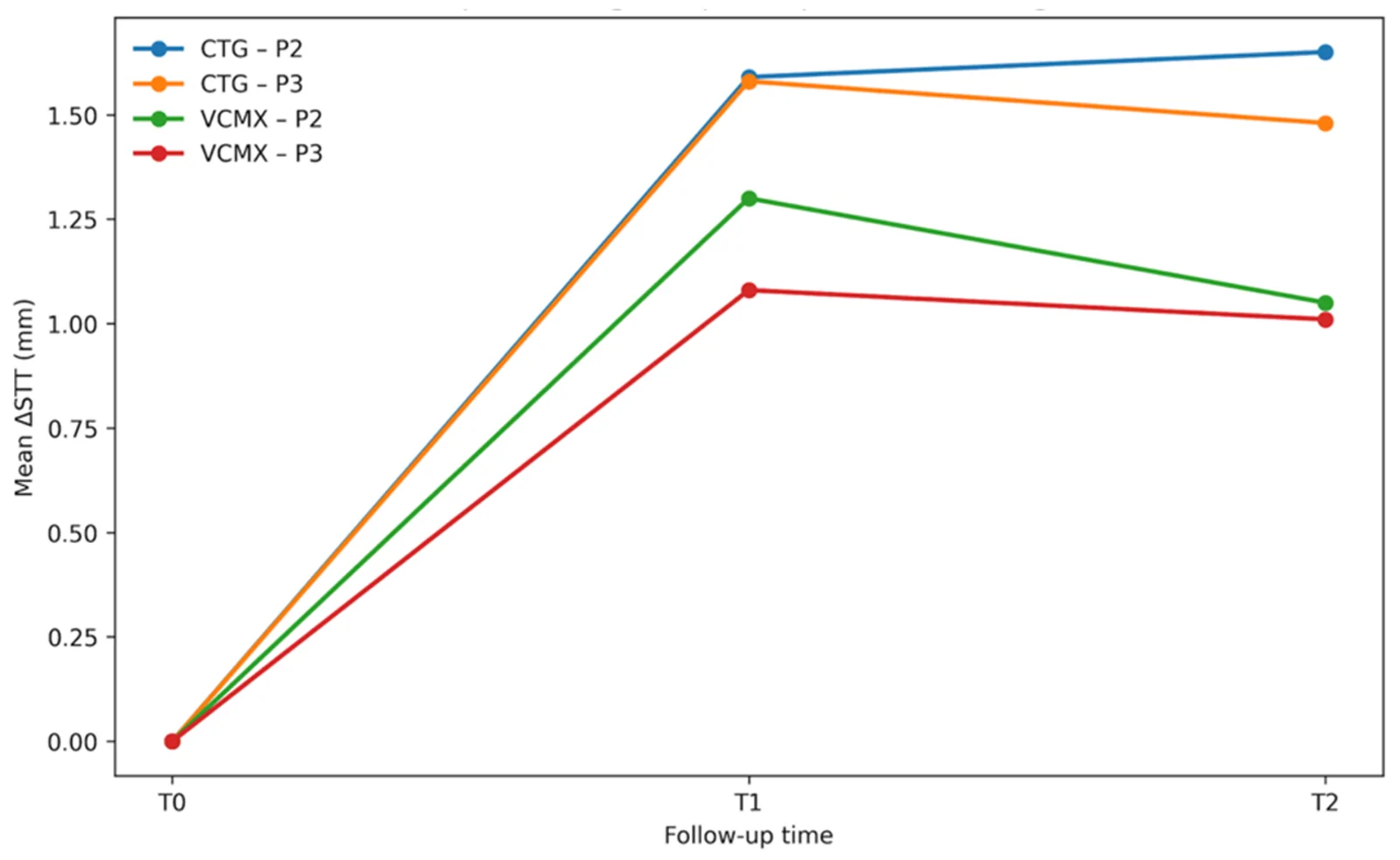
Temporal changes in peri-implant soft tissue thickness.

The temporal dynamics of peri-implant soft tissue response demonstrated a temporal pattern, with the majority of tissue gain occurring during the early healing phase (T0–T1), followed by minimal changes or slight reductions during the late phase (T1–T2) across all groups and measurement sites, Figure 2.

Early gains ranged approximately between 1.0 and 1.6 mm, with consistently higher values observed in the CTG group compared with VCMX. In contrast, late-phase changes were minimal, generally remaining within ±0.2 mm.

Mean changes in soft tissue thickness (ΔSTT) at baseline (T0), early follow-up (T1), and final follow-up (T2) at measurement points P2 and P3 in the CTG and VCMX groups were measured. Most tissue gain occurred during the early healing phase (T0–T1), followed by minimal changes or slight reductions during the late phase (T1–T2).

### 3.3. Relative Soft Tissue Gain (ΔSTT_relative)

**Figure 3:**
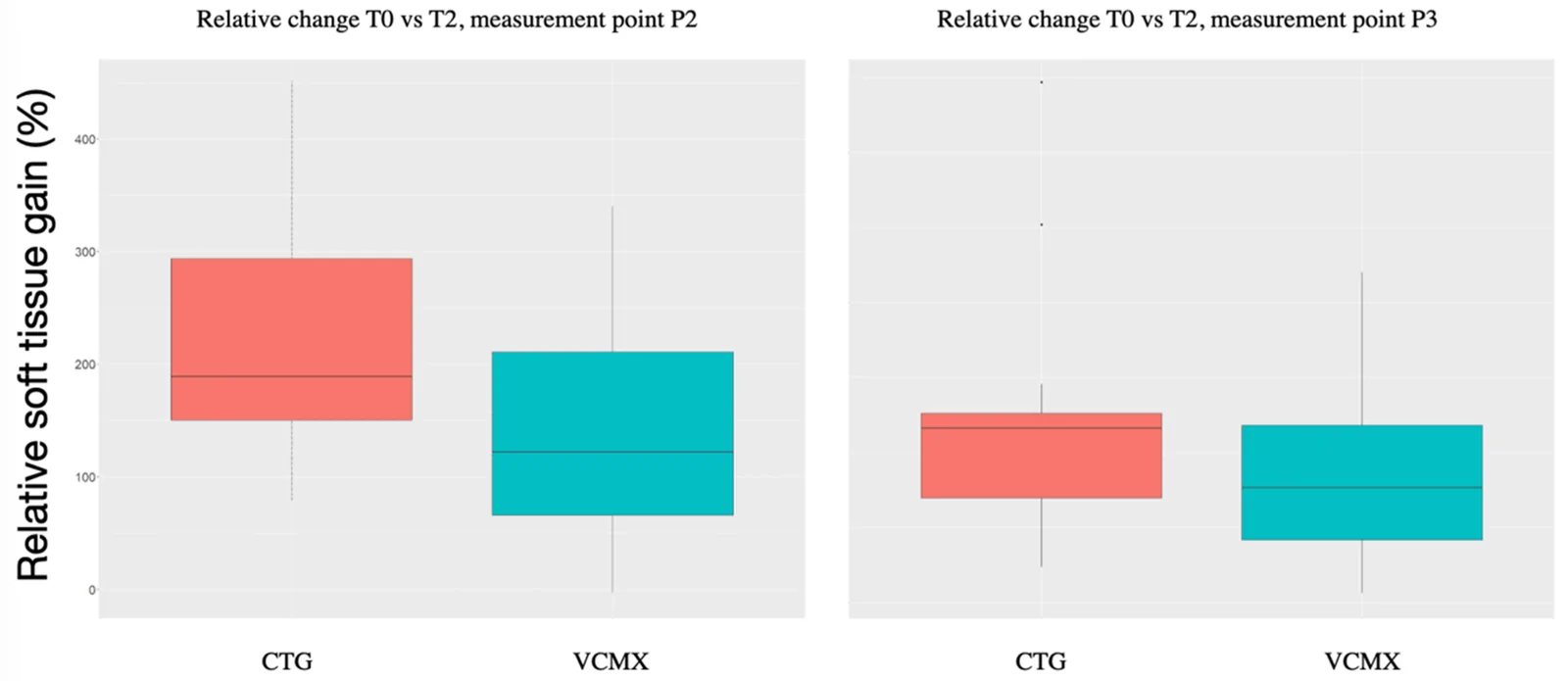
Distribution of relative soft tissue gain (ΔSTT_relative). Boxplots illustrating the distribution of relative soft tissue gain (%) at 12 months (T2–T0) in the CTG and VCMX groups at measurement sites P2 (mid-buccal) and P3 (mucogingival junction).

Both augmentation approaches resulted in marked relative increases in peri-implant soft tissue thickness, Figure 3.

At the final follow-up (T2), mean relative soft tissue gain reached 225% and 236% at P2 and P3 in the CTG group, and 147% and 174% in the VCMX group.

A wide variability in relative response was observed across sites. Relative changes between T1 and T2 were small and did not materially alter the overall 12-month response pattern.

### 3.4. Baseline-Associated Response

The relationship between baseline soft tissue thickness (STT) and subsequent tissue gain was evaluated for both absolute and relative outcomes.

Pearson correlation analyses showed no statistically significant association between baseline STT and absolute 12-month gain in any treatment-group and measurement-site combination (*r* = −0.278 to −0.433; all nominal *p* ≥ 0.082).

In contrast, a statistically significant inverse correlation was observed between baseline STT and relative soft tissue gain (ΔSTT_relative) across all groups and measurement sites. Correlation coefficients ranged from −0.564 to −0.815 (*p* < 0.05).

**Figure 4:**
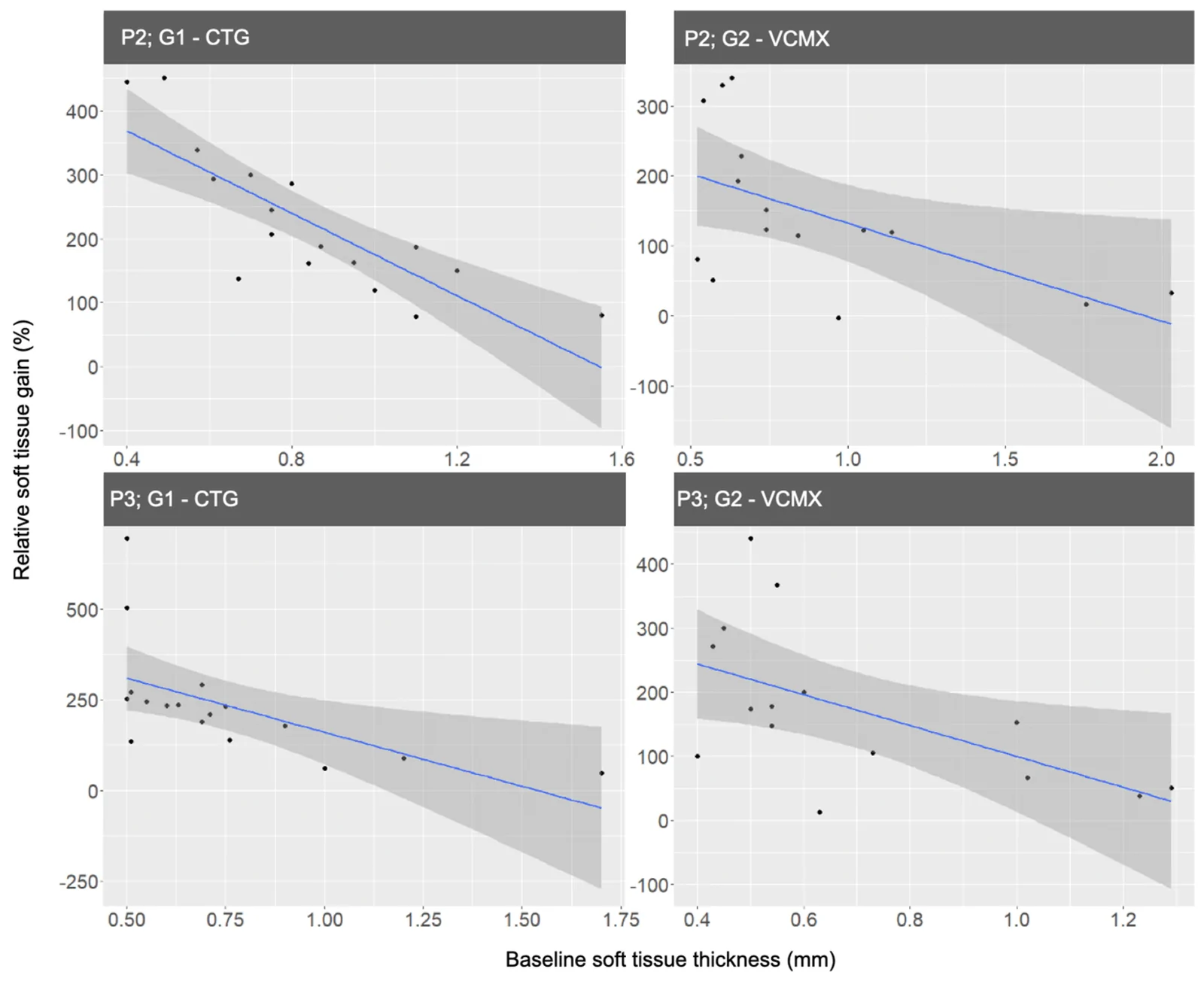
Relationship between baseline soft tissue thickness and relative soft tissue gain (ΔSTT_relative).

This relationship is illustrated in Figure 4, which shows the distribution of relative tissue gain across baseline values.

**Figure 5:**
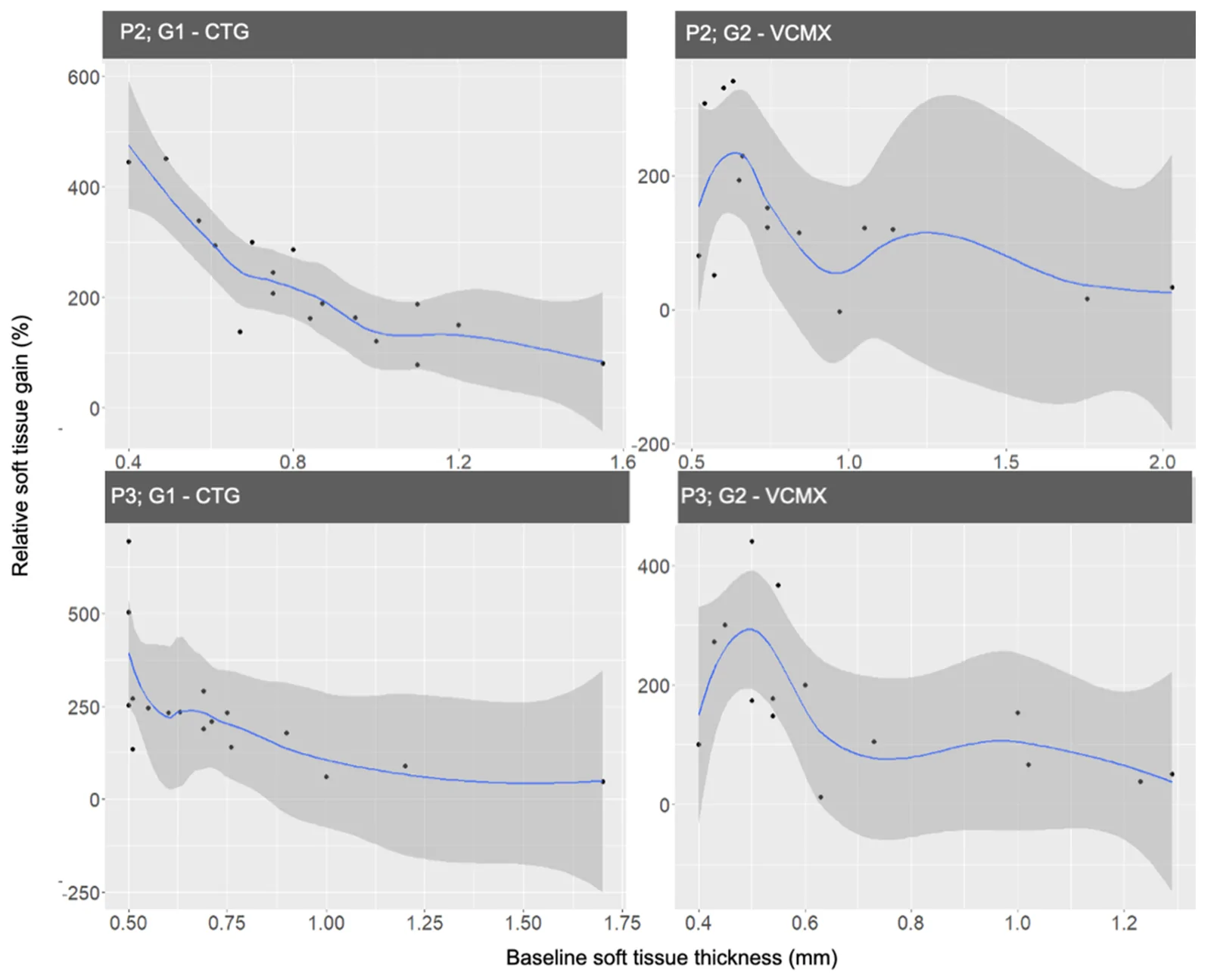
Exploratory LOESS-smoothed relationship between baseline soft tissue thickness and relative soft tissue gain (ΔSTT_relative). Exploratory LOESS curves illustrating the relationship between baseline STT and relative gain at measurement sites P2 and P3 in the CTG and VCMX groups. The curves suggest greater relative gain at lower baseline values and an attenuation of relative gain with increasing baseline thickness. LOESS smoothing was used for descriptive visualization only; no formal test of nonlinearity or threshold effect was performed.

To further characterize this association, locally weighted regression (LOESS) was used as an exploratory visualization (Figure 5). The curves suggested greater relative gain at lower baseline STT values and an attenuation of relative gain with increasing baseline thickness. However, no formal test of nonlinearity or threshold effect was performed, and these patterns should be interpreted cautiously given the limited subgroup sample sizes.

In the exploratory multivariable analysis, baseline STT remained associated with relative tissue gain. Detailed correlation coefficients are presented in Table 2.

Scatter plots illustrating the association between baseline soft tissue thickness (STT at T0) and relative soft tissue gain (ΔSTT_relative) at measurement sites P2 and P3 in the CTG (G1) and VCMX (G2) groups. A significant inverse relationship was observed across all groups and sites, indicating greater relative gain in sites with lower baseline STT.

### 3.5. Exploratory Categorical Analysis

As an exploratory analysis, baseline soft tissue thickness was additionally categorized using a 1 mm threshold (≤1 mm vs. >1 mm) based on the periodontal phenotype classification proposed by Jepsen et al. [20]. At P2, baseline STT was ≤1 mm in 24 sites (CTG, *n* = 13; VCMX, *n* = 11) and >1 mm in 8 sites (CTG, *n* = 3; VCMX, *n* = 5). At P3, the corresponding numbers were 27 (CTG, *n* = 14; VCMX, *n* = 13) and 5 sites (CTG, *n* = 2; VCMX, *n* = 3), respectively.

For absolute soft tissue gain (ΔSTT_absolute), the augmentation method showed a significant effect (H = 6.684, *p* = 0.010), whereas baseline phenotype was not significant (H = 1.076, *p* = 0.300), with no treatment group × phenotype interaction (H = 0.0003, *p* = 0.987). These findings were supported by the robust two-way median analysis (group: Q = 6.014, *p* = 0.014; phenotype: Q = 0.657, *p* = 0.418; interaction: Q = 0.833, *p* = 0.361).

For relative soft tissue gain (ΔSTT_relative), baseline phenotype showed a significant effect (H = 7.403, *p* = 0.007), with thinner tissues (≤1 mm) demonstrating greater proportional increases compared with thicker tissues (>1 mm). The treatment-group effect was not significant (H = 3.000, *p* = 0.083), and no treatment group × phenotype interaction was identified (H = 0.048, *p* = 0.826). The robust two-way median analysis showed the same overall pattern (group: Q = 2.406, *p* = 0.121; phenotype: Q = 5.714, *p* = 0.017; interaction: Q = 0.411, *p* = 0.521).

These results were confirmed in non-parametric two-way analyses.

**Figure 6:**
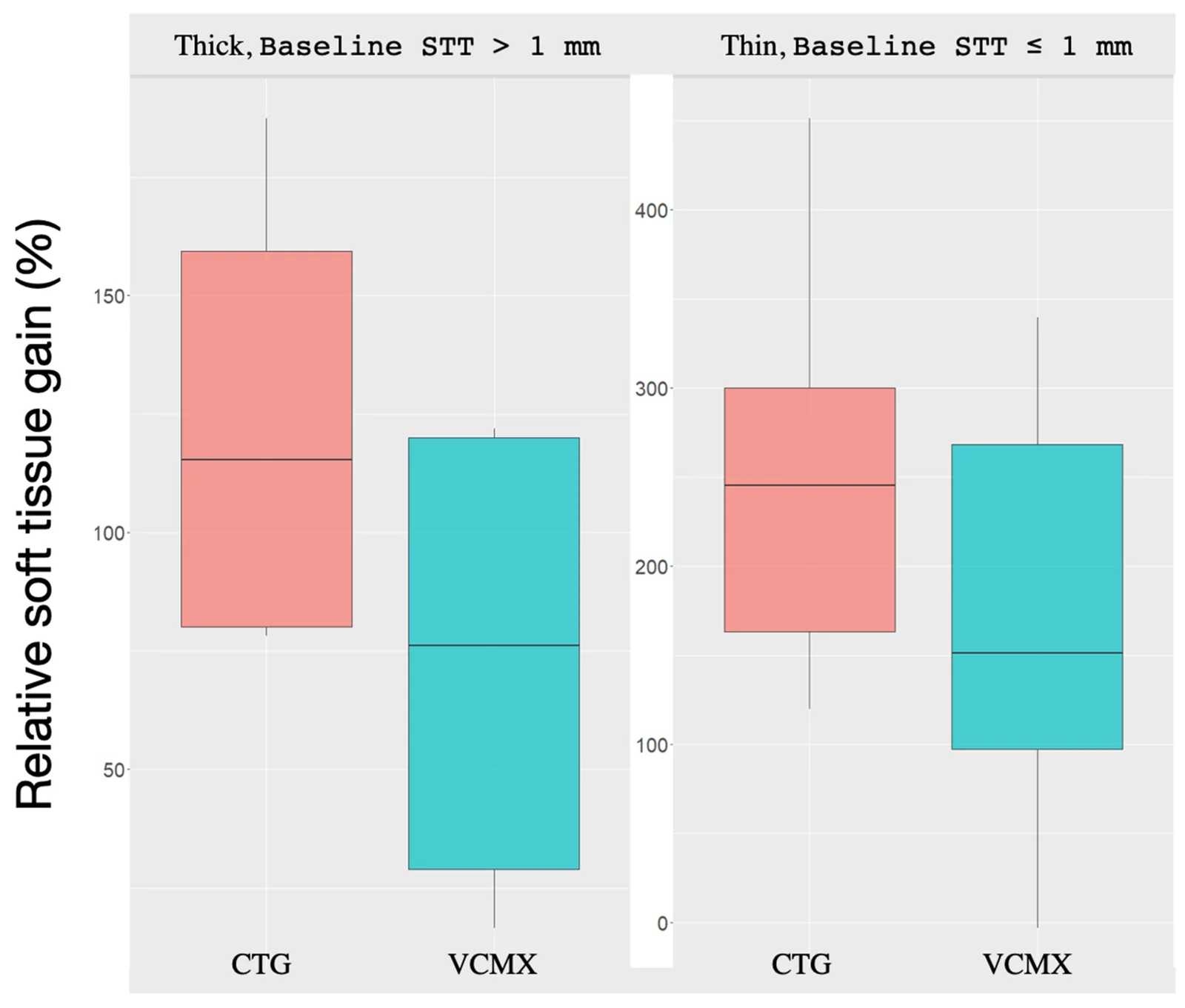
Relative soft tissue gain according to baseline phenotype.

Boxplots illustrating the distribution of relative soft tissue gain (ΔSTT_relative) at 12 months (T2–T0) in sites with baseline soft tissue thickness ≤1 mm and >1 mm, categorized according to Jepsen et al., are presented in Figure 6. Differences between phenotype groups are shown, with no significant interaction between baseline phenotype and treatment group.

### 3.6. Comparison Between Measurement Sites

To evaluate whether the measurement site influenced treatment outcomes, differences in soft tissue gain between P2 and P3 were analyzed.

No significant differences were observed between measurement sites. Paired comparison of absolute soft tissue gain from T0 to T2 showed no statistically significant difference between P2 and P3 (mean paired difference = 0.11 mm, *p* = 0.25).

### 3.7. Procedure Efficiency According to Material Type

The efficiency of soft tissue augmentation, defined as the ratio of absolute tissue gain to graft thickness (ΔSTT/graft thickness), was evaluated.

The mean thickness of CTG grafts harvested from the palate was 1.18 ± 0.17 mm, whereas VCMX grafts had a standardized thickness of 3 mm.

Higher efficiency values were observed for CTG compared to VCMX at both measurement sites. At P2, mean efficiency values were 1.50 ± 0.47 for CTG and 0.35 ± 0.21 for VCMX. At P3, corresponding values were 1.37 ± 0.72 and 0.33 ± 0.19, respectively. The differences between groups were statistically significant at both measurement sites (*p* < 0.001) (Table 3).

Within the CTG group, graft thickness was not significantly correlated with absolute T0–T2 gain at P2 (Pearson *r* = −0.057, *p* = 0.833) or relative T0–T2 gain at P2 (*r* = 0.078, *p* = 0.774).

### 3.8. Multivariable Regression Analysis

Multivariable linear regression models were used to evaluate the adjusted associations of augmentation material, measurement site, and baseline STT with absolute and relative T0–T2 soft tissue gain.

In the model for absolute soft tissue gain, VCMX was associated with lower adjusted gain compared with CTG (β = −0.589, *p* < 0.001), and higher baseline STT was associated with lower absolute gain (β = −0.599, *p* = 0.005). The adjusted difference between P3 and P2 was not statistically significant in the conventional linear regression model (β = −0.199, *p* = 0.154).

In the model for relative soft tissue gain, VCMX was associated with lower proportional gain compared with CTG (β = −78.99, *p* = 0.003), and higher baseline STT was inversely associated with relative gain (β = −221.95, *p* < 0.001). The adjusted difference between P3 and P2 was not statistically significant (β = −14.57, *p* = 0.576).

Median regression was additionally performed as a robust sensitivity analysis. The inverse associations with VCMX and baseline STT remained statistically significant. However, the association between measurement site and absolute gain differed between the conventional and median regression models and should therefore be interpreted cautiously. Complete results of the conventional linear regression models are presented in Table 4.

Both models were based on 64 site-specific observations from 32 implant sites. For the absolute-gain model, R^2^ = 0.332, adjusted R^2^ = 0.298, F(3,60) = 9.927, *p* < 0.001. For the relative-gain model, R^2^ = 0.431, adjusted R^2^ = 0.403, F(3,60) = 15.18, *p* < 0.001.

The models included augmentation material, measurement site, and baseline STT as explanatory variables. P2 and P3 represented anatomically distinct measurement locations and were retained as separate site-specific observations.

## 4. Discussion

### 4.1. Principal Findings

The present study does not aim to re-evaluate treatment efficacy, which has been addressed in the parent randomized clinical trial, but rather to explore baseline-associated patterns of tissue gain. Peri-implant soft tissue augmentation resulted in a measurable increase in tissue thickness, with most of the gain occurring during the early healing phase. Both CTG and VCMX were effective, although CTG was associated with greater absolute and relative tissue gain.

A key finding of this analysis is that baseline soft tissue thickness was associated with relative soft tissue gain. Baseline STT was inversely associated with relative gain, although this association is partly driven by the mathematical dependence of relative gain on baseline values. While absolute tissue gain showed no consistent association with baseline values in univariable analyses, relative gain demonstrated a clear inverse relationship, with greater proportional increases observed in sites with thinner tissues.

These exploratory findings suggest that baseline tissue conditions may be associated with variability in augmentation outcomes. However, no treatment-by-phenotype interaction was detected, and the present analysis does not demonstrate that baseline phenotype should determine the selection of CTG or VCMX.

### 4.2. Clinical Interpretation of Baseline-Associated Response

Our results showed that baseline soft tissue thickness was inversely associated with relative soft tissue gain, with greater proportional increases observed at sites with lower baseline thickness. This observation is consistent with previous evidence indicating that peri-implant and periodontal tissue outcomes may be associated with baseline phenotype and tissue characteristics [12,13,21,22,23,24].

In the present study, baseline soft tissue thickness was additionally categorized using a 1 mm cut-off based on the periodontal phenotype classification proposed by Jepsen et al. [20]. Importantly, this exploratory categorization applied to facilitate comparisons between relatively thinner and thicker baseline facial tissues. Recent histology-based evidence suggests that this dichotomous classification may oversimplify the biological variability of gingival thickness. Sabri et al. [25] proposed a refined cut-off value of approximately 1.18 mm, demonstrating improved diagnostic accuracy for distinguishing thin versus thick gingiva.

These observations support the concept that soft tissue phenotype should be interpreted as a continuous rather than strictly dichotomous variable. In line with this, the present study showed an inverse association between baseline thickness and relative tissue gain, with relative gain progressively decreasing as baseline thickness increased. These findings are consistent with previous evidence showing that connective tissue grafts remain the most effective approach for increasing mucosal thickness, although the magnitude of gain is limited and influenced by post-surgical remodeling. Network meta-analysis data by Cafasso et al. [15] indicate that connective tissue grafts achieve a mean mucosal thickness gain of approximately 0.94 mm, compared to approximately 0.45 mm for collagen matrices. Similarly, Tavelli et al. [26] reported greater gains for connective tissue grafts and acellular dermal matrices compared to collagen matrices.

Clinical studies also show that soft tissue augmentation is followed by dynamic post-surgical remodeling, characterized by an initial increase in tissue volume and a subsequent partial reduction over time [27,28,29]. Greater percentage gains at sites with lower baseline thickness should be interpreted cautiously because relative change is mathematically influenced by the smaller baseline denominator and does not necessarily indicate greater soft tissue response.

Overall, these findings indicate an inverse association between baseline soft tissue thickness and relative gain. Although the present secondary analysis focused on baseline-associated soft tissue remodeling, its findings should be interpreted in the context of the parent randomized controlled trial [16], which demonstrated minimal marginal bone loss, stable implant survival, and no biological or prosthetic complications during the 12-month follow-up. Therefore, the observed tissue changes occurred under clinically stable peri-implant conditions.

### 4.3. Comparison with Previous Studies

In our study, greater relative tissue gain at the final follow-up was observed in the CTG group compared to the collagen matrix group. At P2, mean relative gain reached approximately 225% in the CTG group versus 147% in the collagen matrix group. The corresponding absolute gain reported in the parent RCT was 1.65 mm and 1.05 mm, respectively. Similar trends were observed at P3.

These findings are consistent with previous evidence demonstrating that connective tissue grafts show superior effectiveness compared to collagen matrices [11,27,30,31]. This difference may be related to soft tissue response variations in tissue integration and remodeling processes, including early vascularization and fibroblast activity, which are known to influence the behavior and integration of collagen-based biomaterials during healing [32]. Unlike autogenous CTG, VCMX functions as a collagen scaffold that requires host-cell infiltration and neovascularization for tissue integration. Its progressive remodeling and degradation during healing may therefore partly contribute to the lower augmentation efficiency observed with VCMX [32].

Previous studies have also highlighted the importance of peri-implant soft tissue characteristics, particularly the association between thin mucosa and increased susceptibility to marginal bone loss and reduced tissue stability [33,34,35,36].

However, most available studies have focused primarily on absolute changes in tissue thickness, without evaluating proportional change relative to baseline. The present exploratory analysis identified an inverse association between baseline soft tissue thickness and relative gain. This finding should be interpreted together with absolute gain, as percentage change is partly influenced by the baseline denominator.

### 4.4. Implications for Personalized Treatment Planning

Previous evidence suggests that peri-implant soft tissue characteristics play a critical role in maintaining peri-implant tissue stability. Increased mucosal thickness has been associated with reduced early marginal bone loss, while favorable transmucosal profile configurations have been shown to improve peri-implant hard tissue outcomes [8,37]. Together, these findings support the concept that peri-implant phenotype characteristics represent biologically relevant determinants of treatment outcomes.

An important observation of the present study is the discrepancy between absolute and relative measures of soft tissue gain. While absolute increases in tissue thickness differed moderately between augmentation methods, relative gains revealed more pronounced differences.

Relative soft tissue gain provides a complementary description of proportional tissue change that is not captured by absolute measurements alone. In particular, sites with lower baseline soft tissue thickness showed greater proportional increases, whereas sites with thicker baseline tissues demonstrated a more limited relative response. However, greater percentage gain at sites with lower baseline STT is partly influenced by the smaller baseline denominator and does not necessarily indicate greater soft tissue responsiveness or clinical benefit. The extent of augmentation should primarily be guided by the absolute tissue deficiency and the desired final soft tissue dimensions.

These findings indicate that treatment outcomes should be considered in relation to baseline tissue conditions rather than applied uniformly across implant sites. From a clinical perspective, even modest absolute tissue thickening may be particularly relevant at sites with a thin baseline phenotype, while the larger relative gain observed in such sites should be interpreted with consideration of its mathematical dependence on baseline STT.

Conversely, in sites presenting with an already thick soft tissue phenotype, the objective of augmentation may not necessarily be to maximize tissue volume. Excessive tissue thickening may compromise flap adaptation and increase tissue tension, potentially affecting wound stability. In such situations, less extensive augmentation procedures, thinner autogenous grafts, or less bulky soft tissue substitutes may represent a more appropriate approach to achieve biologically favorable tissue dimensions while avoiding excessive soft tissue bulk. Therefore, the selection of the augmentation material should be guided not only by its regenerative potential but also by the baseline tissue phenotype and the desired final tissue thickness.

From a clinical perspective, connective tissue grafts remain the most predictable approach for increasing mucosal thickness, particularly in cases requiring greater tissue augmentation [10,15].

The different patterns observed for absolute and relative gain indicate that both measures should be considered when interpreting augmentation outcomes.

These findings support a more individualized approach to peri-implant soft tissue management, where baseline tissue characteristics may guide both treatment selection and expected clinical outcomes, in line with contemporary concepts of personalized and risk-based therapy [38,39].

### 4.5. Limitations

Several limitations of the present study should be considered. First, the analysis represents a secondary evaluation of an existing dataset and was not originally designed to assess baseline-associated factors.

Second, the sample size was limited, and the analysis was restricted to 12 months follow-up.

Third, two patients contributed more than one implant site. In both cases, the sites were spatially distinct and were assigned to different treatment groups; thus, no patient contributed more than one implant site within the same treatment arm. Although residual patient-level correlation between these sites cannot be completely excluded, the potential impact of patient-level clustering was limited by the very small number of patients contributing multiple sites. Repeated P2/P3 measurements over time within individual implants were accounted for using a mixed-effects model with implant ID as a random intercept. In the exploratory pooled multivariable analyses, P2 and P3 were retained as separate site-specific observations; therefore, residual within-implant correlation cannot be completely excluded and should be considered when interpreting these analyses.

Fourth, the relative gain is mathematically influenced by the baseline denominator and may therefore emphasize proportional changes at sites with lower baseline STT. Accordingly, relative gain should be interpreted together with absolute gain and not as direct evidence of greater soft tissue response.

Finally, residual variability related to patient- and site-specific factors may have influenced the observed outcomes.

Overall, the present findings should be interpreted as exploratory and hypothesis-generating.

## 5. Conclusions

Peri-implant soft tissue augmentation resulted in a measurable increase in mucosal thickness, with connective tissue grafts demonstrating greater absolute and relative tissue gain than volume-stable collagen matrices.

Baseline soft tissue thickness showed an inverse association with relative tissue gain; however, this relationship is partly influenced by the mathematical dependence of relative gain on baseline values.

These findings indicate that the outcome of soft tissue augmentation can be potentially influenced by baseline tissue conditions and support a phenotype-informed approach to treatment planning, taking into account differences in tissue responsiveness.

Given the exploratory design and the mathematical dependence of percentage change on baseline values, these findings should be interpreted as hypothesis-generating.

## Figures and Tables

**Table 1:** Baseline characteristics of the study population.

Variable	CTG (*n* = 16)	VCMX (*n* = 16)
Age (years), mean ± SD	37.9 ± 8.8	37.7 ± 8.5
Baseline STT (mm)		
P2 (mid-buccal)	0.84 ± 0.29	0.90 ± 0.45
P3 (MGJ level)	0.75 ± 0.31	0.69 ± 0.30

STT—soft tissue thickness; P2—mid-buccal point; P3—mucogingival junction.

**Table 2:** Correlation between baseline soft tissue thickness (STT) and tissue gain.

Outcome	Site	Group	*n*	Pearson *r*	95% CI	*p*-Value
Absolute gain	P2	CTG	16	−0.383	−0.74 to 0.14	0.130
Absolute gain	P2	VCMX	16	−0.372	−0.73 to 0.15	0.172
Absolute gain	P3	CTG	16	−0.433	−0.76 to 0.08	0.082
Absolute gain	P3	VCMX	16	−0.278	−0.68 to 0.25	0.316
Relative gain	P2	CTG	16	−0.815	−0.93 to −0.54	<0.001
Relative gain	P2	VCMX	16	−0.564	−0.83 to −0.09	0.029
Relative gain	P3	CTG	16	−0.591	−0.84 to −0.13	0.012
Relative gain	P3	VCMX	16	−0.571	−0.83 to −0.11	0.026

**Table 3:** Graft thickness and efficiency of soft tissue augmentation.

Parameter	CTG (*n* = 16)	VCMX (*n* = 16)	*p*-Value
Graft thickness (mm)	1.18 ± 0.17	3.00 (fixed)	—
Efficiency (P2)	1.50 ± 0.47	0.35 ± 0.21	<0.001
Efficiency (P3)	1.37 ± 0.72	0.33 ± 0.19	<0.001

Values are presented as mean ± SD unless otherwise indicated. Efficiency was calculated as ΔSTT/graft thickness.

**Table 4:** Multivariable linear regression models for absolute and relative soft tissue gain.

Predictor	ΔSTT_absolute β (SE)	95% CI	*p*-Value	ΔSTT_relative β (SE)	95% CI	*p*-Value
VCMX vs. CTG	−0.589 (0.135)	−0.859 to −0.320	<0.001	−78.99 (25.32)	−129.64 to −28.34	0.003
Baseline STT	−0.599 (0.204)	−1.007 to −0.191	0.005	−221.95 (38.35)	−298.66 to −145.24	<0.001
P3 vs. P2	−0.199 (0.138)	−0.474 to 0.077	0.154	−14.57 (25.91)	−66.40 to 37.26	0.576

## Data Availability

The datasets analyzed during the current study are not publicly available due to ethical and privacy restrictions but are available from the corresponding author on reasonable request.
